# State-of-the-arts methods for studying factors driving the utilization of open science resources

**DOI:** 10.1016/j.mex.2024.103125

**Published:** 2024-12-21

**Authors:** Le Thi Tuyet Trinh, Nguyen Thi Thu Hang, Le Minh Cuong, Ngo Van Dinh, Hoang Khanh Linh, Do Thi Trinh, Nguyen Thuy Phuong Tram, Ho Nguyen, Manh-Tung Ho

**Affiliations:** a Dong Thap University, 870000 Cao Lanh City, Dong Thap, Viet Nam; b Thai Nguyen University of Agriculture and Forestry, Thai Nguyen University, Thai Nguyen, Vietnam; c School of Culture – Department of Training – Ministry of Public Security, Thai Nguyen, Vietnam; d VNU University of Education, Vietnam National University, Hanoi, Vietnam; e Vietnam Journal of Education - Vietnam Journal of Education - Ministry of Education and Training. Hanoi, Vietnam; f Thai Nguyen University of Education. No. 20 Luong Ngoc Quyen Street, Thai Nguyen City, Thai Nguyen Province, Vietnam; g Ductrong Highschool-Lam Dong Province. No. 320 National Highway 20, Lien Nghia, Duc Trong, Lam Dong Province, Vietnam; h Institute of Landscape Ecology, University of Münster, 48149 Münster, Germany; i Centre for Interdisciplinary Social Research, Phenikaa University, Yen Nghia, Ha Dong, 100803, Hanoi, Vietnam

**Keywords:** Open science, Technological acceptance, Value-filtering, Motivations, Self-Efficacy, Integrated Analytical Framework for Open Science Resource Utilization

## Abstract

The open science movement aims to democratize essential scientific values, including *rigor, transparency, availability, and replicability*. However, open science resources are still often underutilized. A comprehensive evaluation of the factors influencing the use of these resources is urgently needed. This evaluation not only supports evidence-based policymaking to help researchers thrive in a highly competitive academic environment but also prevents misunderstandings and confusions related to emerging scientific practices. This article presents a survey-based framework for understanding the factors that affect the ability to utilize open science resources. To achieve this, we will integrate relevant factors such as capabilities, attitudes, perceived costs and benefits, cultural and institutional influences, and alignment of values into our analytical framework. These factors are derived from significant theories that have been applied to study the acceptance and use of information systems: the Theory of Planned Behavior, Self-Determination Theory, the Unified Theory of Technology Use and Acceptance, Self-Efficacy Theory, and the value-filtering model known as Mindsponge Theory.We will briefly introduce the key elements of these frameworks and discuss their applications in the context of utilizing open science resources as well as in the broader context of innovation adoption. Furthermore, we argue that elements from these models significantly overlap and complement one another. Therefore, to thoroughly evaluate the factors affecting the utilization of open science resources, it is essential to coherently combine aspects from these models into a unified analytical framework.

## Review Highlights

• This study first argues that the utilization of open science resources can be conceptualized in two ways: the process of accepting a new information system and technology, and the process of filtering and adapting new values into an existing value system.
• It then reviews commonly used methods, namely the Mindsponge model of value-filtering, the Technology Acceptance Model, the Unified Theory of Acceptance and Use of Technology, Self-Determination Theory, and Self-Efficacy Theory.
• We propose a questionnaire that integrates factors from these methods and presents sample questions for two categories of subjects: university students and university lecturers/staff. Some discussion on applications of statistical methods is also provided.

## Graphical abstract

**Figure:**
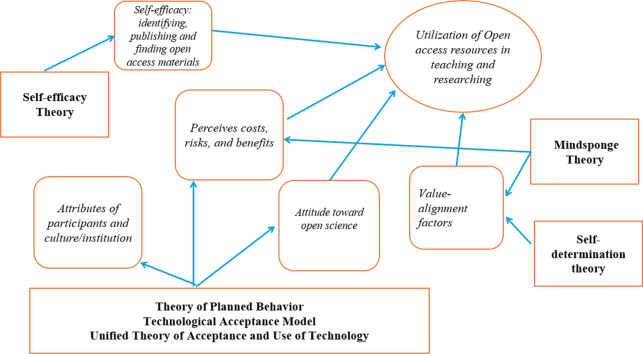

Specifications table

**Table:** 

Subject area:	*Psychology*
More specific subject area:	*Studies of acculturation; Studies of technological adoption*
Name of the reviewed methodology:	*Mindsponge, Technology Acceptance Model; Unified Theory of Acceptance and Use of Technology; Self-Determination Theory; Self-Efficacy Theory*
Keywords:	*Open Science; technological acceptance; Information and communication technologies; value-filtering; mindsponge*
Resource availability:	*NA*
Review question:	*-Which factors drive the utilization of open science resources?* ***-****Viewing utilization of open science resources as acceptance and use of new information technologies and as value-filtering process, how can we theorize about factors that determine open science resources utilization?*

## Background

There is little doubt that the Open Science initiative has forever changed scholarly communication. The concept of Open Access was first introduced through three public statements in the 2000s, including the Budapest Open Access Initiative (2001), the Bethesda Proclamation on Open Access Publishing (2003), and the Berlin Declaration on Open Access to Knowledge in the Sciences and Humanities (2003). Since then, there has been massive growth in the number of open access (OA) journals around the world. For example, according to a study by Pandita and Singh (2022) [1], between 2002 and 2021, the average annual growth rate of OA journals was around 51.46 %, resulting a remarkable rise from only 22 OA journals were indexed in Directory of Open Access Journals in 2002 to 16,589 OA journals indexed in 2021 [1].

To researchers around the world, especially those from the Global South and early career researchers, Open Science presents unprecedented opportunities for accessing high-quality research information at little to no cost. It is often reported that open science movement has contributed to the improved *rigor, transparency, replicability,* and *availability* of science around the world [2,3]. Consequently, in recent years, research studies on factors influencing the utilization of open science resources in academia have gained traction [[4], [5], [6], [7]]. To further elaborate, a large-scale textual analysis by Pinfield (2015) of the peer-reviewed literature since 2010 on the topic of OA showed that the discourse on OA had moved from whether it should happen at all to how to make it work [8].

On one hand, the rising research interest in this topic is driven by the practical need to improve scientific standing and knowledge transfer around the world. Researchers and science policymakers from various backgrounds - from the hard sciences to humanities, from Western countries to the Global South - are looking for ways to maximize the gains in capabilities and reduce the cost of accessing scientific resources from the Open Science movement and its increasingly widely available resources such as data, methods, open access journal articles and books, etc. [5,7,9]. Many studies show that stakeholders of higher education know somewhat about open science resources, yet unequivocally under-utilizing the valuable sources [4,6]. For example, a mixed-method study of the Malaysian Comprehensive Public Universities in 2021 revealed, the term Open Science was still new to most participants, while the public universities partially engaged with it [10]. Thus, understanding factors that influence the utilization of open science resources has become an urgent matter to find ways to increase the utilization of these low-cost resources, to not only improve scientific capability, but also to survive and stand out in a harshly competitive world.

On the other hand, it is widely acknowledged, at least among Global South scholarly communities, that many researchers lack the skills and knowledge to fully capitalize on the Open Science movement. Worryingly, many individuals have fallen victim to predatory practices disguised as Open Science. Additionally, numerous reports indicate feelings of unease and anxiety associated with the adoption of this entirely new approach to scientific research [11]. In the best-case scenarios, this has led to confusion regarding emerging scientific practices; in the worst cases, it has resulted in unwanted outcomes such as interpersonal conflicts, outright malpractices and fraud committed under the guise of the Open Science movement [12].

This background necessitates a comprehensive evaluation of factors that influence the utilization of open science resources, because they not only support evidence-based policymaking but also prevent future harm caused by misunderstanding and confusion related to the emerging practices.

While researchers have started to unpack factors driving the utilization of open science resources from many angles, most reviews and empirical studies have significantly overlapped scientific hypotheses and constructs; and the evidence is far from conclusive. For example, in a comprehensive review of drivers and inhibitors of open data sharing and using practices from 32 studies published between 2004 and 2015, Zuiderwijk et al. (2020) identified eleven categories including: ‘the researcher's background’, ‘requirements and formal obligations’, ‘personal drivers and intrinsic motivations’, ‘facilitating conditions’, ‘trust’, ‘expected performance’, ‘social influence and affiliation’, ‘effort’, ‘the researcher's experience and skills’, ‘legislation and regulation’, and ‘data characteristics.’ [6]. In another study, Zia and Nazim (2023) examine the use of Open Access resources among faculty members and researchers in North India, the authors find that most of the research subjects are aware of the OA resources, but do not utilize them enough. The analysis shows critical determinants including internet self-efficacy, awareness and attitude toward OA, accessibility, trustworthiness, professional recognition, academic reward, altruism, mandates and culture, and individual traits [4]. However, the literature appears to lack a comprehensive overview of the methods for studying utilization of open science resources. Therefore, this study attempts to fill the gap by proposing a synthesis of Theory of Planned Behavior [13,14], self-determination theory [15], Unified Theory of Technology Use and Acceptance [16,17], self-efficacy theory [18], and the value-filtering model called mindsponge [[19], [20], [21]].

## Method details

### The complexity of utilizing open science resources

In this section, we discuss various determinants of open access utilization. Contrary to our intuition would suggest, synthesizing the literature reveals that ease of access and cost-effectiveness do not simply mean a significant increase in the rate of utilization of open science resources. Moreover, simply mandating researchers to publish in open access journals or books or simply introducing students, staff, lecturers, researchers to open science sources might not guarantee a sustained and intelligent engagement with open science resources. We believe the new practice of utilizing open science resources can be conceptualized in two ways.

First, to utilize open science resources, one must have access to information technology, therefore, we can view this behavior as about accepting and adopting new informational systems and practices. Here, this view immediately highlights the importance of behavioral and cognitive factors of whether an individual rates the difficulty (technical and otherwise) in accessing open science, the financial costs involved, the utilities of utilizing open science resources, the risks involved in engaging these new practices, etc. For example, Kankam et al. (2024)’s study on the Ghanaian researchers found that most of them reported feeling the benefits from open access movement, but having trouble with credibility issue and high cost [22]. Similarly, Mangai and Ganesan (2021) showed factors of easy to use and ease to access (all in one place) were the reasons for scholars using open or low-cost resources such as e-books, e-journals, etc., while limited access to computers, delay in downloading and lack of search skills could be the mitigating factors [23]. As such, this view necessitates that we can begin to unpack this problem by looking at theories related to acceptance and use of technology such as Technological Acceptance Model, Unified theory of Acceptance and Use of Technology.

In addition, perceived technical difficulties in utilizing open sciences involve the self-efficacy elements, it also warrants an examination of Self-efficacy theory. For example, Ajegbomogun and Popoola's study on Nigerian scientists revealed that self-efficacy, perceived usefulness, accessibility of Internet resources, and utilization of Internet resources influenced significantly to research productivity of the respondents. And the authors concluded that self-efficacy should be prioritized when recruiting individuals for lecturing positions at universities, because the analyses they fostered an intrinsic drive for knowledge creation and helped cultivate positive attitudes towards advancing research [24].

Second, the open science movement represents, to many institutions and researchers, a new value and a new way of doing science, which means the utilization of open science is fundamentally about adopting new values into existing cultural, organizational, and personal core value system. This process necessarily implicates value changes and conflicts as there are differential levels of beliefs and commitment to the ideal that information should be free among people and communities [25,26]. For example, Caffrey and Gardner (2023) showed that crowdsourced research sharing behaviors are often motivated by utilitarian rationale or ideology in nature. Similarly, a study in Research Policy that examines personal attitudes and principles regarding open data sharing of randomly selected scientists showed this was still an ideal professed but not yet practiced [27]. Hence, this view highlights the importance of considering beliefs, values, and motivations in determining the level of utilization of open science resources [23,27,28]. In turn, this reasoning gives credence to the theories such as self-determination theory and mindpsonge value-filtering model, which emphasized the importance of these factors.

Viewing the practice of utilizing open science resources in these two ways gives us a starting point to review a number of theories and models that will help unpack the factors driving the utilization of open science resources. Based on these two observations, this article seeks to provide a survey-based framework for understanding factors underlying capability of utilization of open science resources. To do so, we combine relevant factors such as capabilities, attitudes, perceived costs and benefits, cultural and institutional factors, and alignment of values to create a analytical framework. These factors are drawn from important theories that have been the Theory of Planned Behavior [13,14], self-determination theory [15], Unified Theory of Technology Use and Acceptance [16,17], self-efficacy theory [18], and the value-filtering model called mindsponge [[19], [20], [21]].

### A brief description and reviews of relevant theories

We will briefly introduce key elements of these frameworks and identify the frameworks’ applications in the context of open science resources utilization and of the larger context in adoption of innovations. Then, we argue that elements from these models greatly overlap and complement each other. Thus, to comprehensively evaluate factors in open science resources utilization, it is critical to combine factors from these models coherently into an analytical framework.

### From the theory of planned behavior to unified theory of acceptance and use of technology

One of the most well-known and well-cited theories for studying factors behind the acceptance and adoption of technological innovations is the Theory of Planned Behavior (TPB) [29]. This theory was developed by social psychologists, most notably Icek Ajzen [13,14], stating: “Being neither capricious nor frivolous, human social behavior can best be described as following along lines of more or less well-formulated plans” [14]. At its core, this theory posits that people's engagement in a given volitional behavior is a function of three belief-based factors: attitudes, subjective norms, and perceived behavioral control [29].

Specifically, attitudes are variables corresponding to subjective evaluations of the researched behavior, which seek to understand an individual's view on the amount of effort, time, and benefits (material or mental) derived from performing such a behavior. Subjective norms measure the perceived social appropriateness of the behavior, often taking the form of questions about how individuals perceive their peers or respected members of their groups would feel if they engage in the studied behavior. Perceived behavioral control measures the subject's confidence in his or her ability to engage in the behavior, which corresponds with a key construct in the Self-efficacy theory.

This theory has achieved considerable success in providing a reasonable account for a wide range of behaviors, such as medical interventions [30], adoption of new technologies such as Non-Fungible Tokens Metaverse [31] or driverless cars [32], and implementation of new practices [4,31], etc. Developed based on the Theory of Planned Behavior, there are at least two very successful theories: the Technology Acceptance Model (TAM) and Unified Thery of Acceptance and Use of Technology. The original Technology Acceptance Model (TAM), first proposed by Fred Davis in 1989, posited that acceptance of a new technology could be primarily accounted for by two major factors: perceived ease of use and perceived usefulness [33]. A decade later, the model was extended with the addition of the subjective norms variable, which is similar to that in the Theory of Planned Behavior (TPB) and measures how a person perceives that important or close others view the studied technology [34].

In the 2000s, TAM, TPB, and Self-Determination Theory (SDT), among others, inspired the next generation of researchers to design the Unified Theory of Acceptance and Use of Technology (UTAUT) [17], which aims to unpack the factors driving the intention to accept and subsequently use a new technology or information system in an organizational setting. In UTAUT, the predictive variables include *performance expectancy, effort expectancy, social influence,* and *facilitating conditions;* and outcome variables include intention to adopt the new technology and actual usage behavior. The four moderating constructs are age, gender, experience and voluntariness of use [17].

As shown in Table 1,clearly, performance expectancy overlaps with TAM and TPB's perceived utilities; effort expectancy corresponds to TAM and TPB's perceived ease of use; social influence corresponds to TAM and TPB's subjective norms; while facilitating conditions were a newly introduced construct, yet it has been included in later extended versions of TAM [[35], [36], [37], [38]]

**Table 1:** Definitions of key constructs in the UTAUT, TAM, TPB.

Theories	Key predictor constructs	Definitions	Corresponds/overlaps
UTAUT	Performance expectancy	the degree to which an individual believes that using the system will help him or her to achieve some gains in performance [17]	TAM and TPB's perceived utilities
	Effort expectancy	“the degree of ease associated with the use of the system” [17]	TAM and TPB's perceived ease of use
	Facilitating conditions	“the degree to which an individual believes that an organisation's and technical infrastructure exists to support the use of the system” [17]	TPB and extended TAM's perceived combability
	Social influence	"the degree to which an individual perceives that important others believe he or she should use the new system" [17]	TAM and TPB's subjective norms

**Fig. 1:**
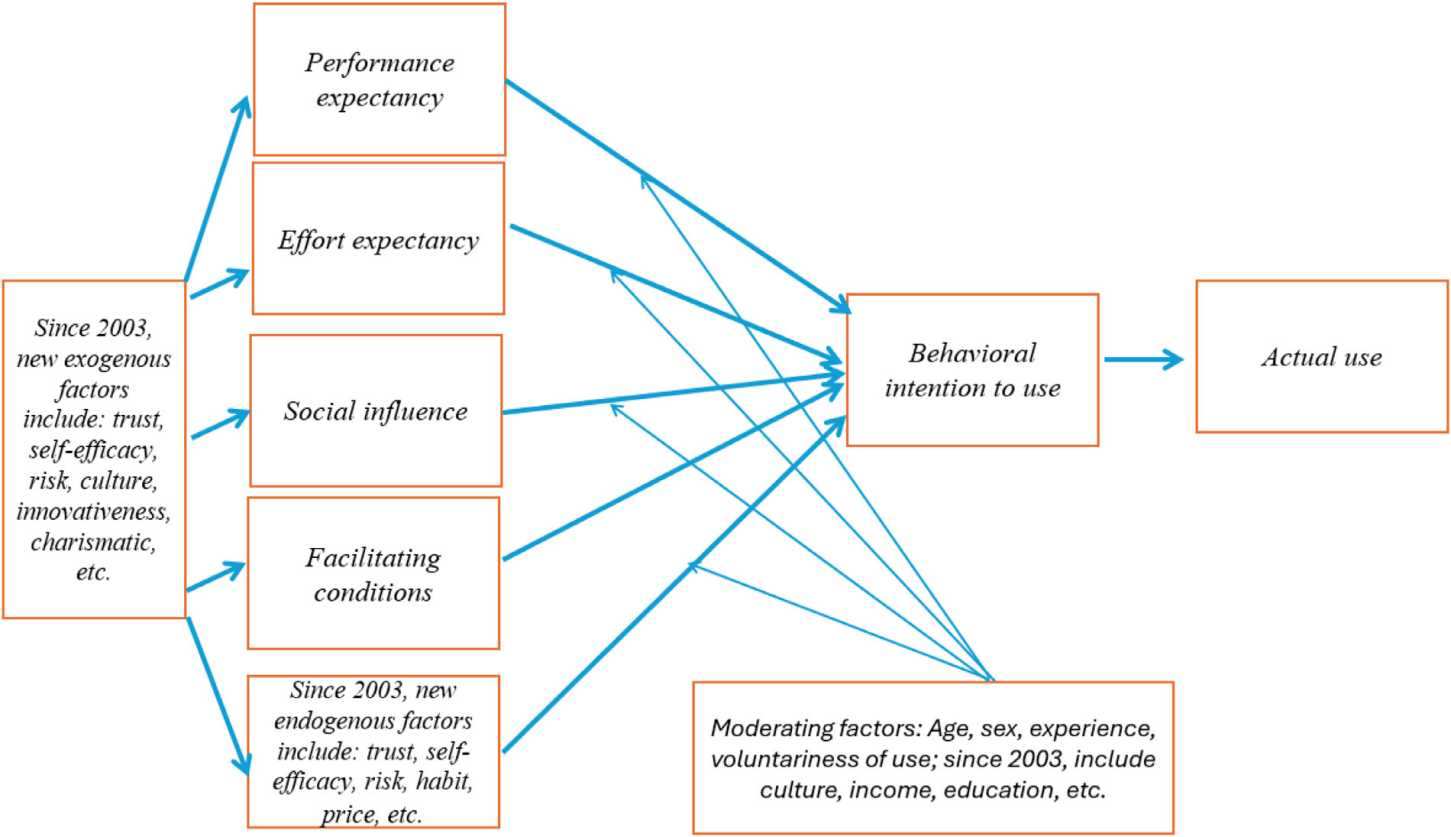
A diagram to summary of the development of the Unified Theory of Acceptance and Use of Technology since 2003 reproduced from the original by Venkatesh et al. (2016) [17].

Fig. 1 summarizes the development of the UTAUT since 2003. Most notably, in 2012, Venkatesh et at [39]. proposed the extended version called UTAUT2, which incorporated three new constructs into UTAUT: hedonic motivation, price value, and habit. In the 2016 review article, Venkatesh et at reviewed applications of the UTAUT/UTAUT2 from 2003 to 2014, and argued for a *multi-level framework* where the UTAUT/UTAUT2 served as the baseline model, subjected to influences from the high-level contextual factors (attributes of the environment, organization, and location) [16] and low-level factors (attributes of users, technologies, and tasks). There was also the events (time) dimension, where the authors recognized the fact that time can change the perceptions of users or groups of the attributes of the studied technology.

Looking at the fattening of the UTAUT as well as the TAM, there are at least two concerns here. Firstly, while it is clear that UTAUT has demonstrated a strong level of flexibility to include many factors [40], the overgrowing of the number of endogenous and exogenous factors as well as moderating mechanisms undermine the ideal of scientific parsimony. Secondly and critically, given that we need to apply the theory to the specific context of open science utilization in higher education, there must be better method of reasoning about which factors to include in the final model.

Yet, the UTAUT and its extended version has yielded great insights, we can have a number of well-educated hypotheses in the case of utilizing open science resources as below:

(i) positive costs-and-benefits evaluations (such as perceived ease of use, perceived utilities, perceived efforts, etc.) can influence level of utilization.
(ii) social appropriateness (other people also express the same attitude) and favorable facilitating conditions (institutional mandates, ease of access, favorable infrastructure, etc.) can drive a higher level of utilization of open science resources.
(iii) Attribute of users (e.g., age, sex, years of research experience, etc., place of residence) can also influence levels of open science resources utilization whether by moderating effects or direct effects.

### Motivation-related theories: Self-determination theory and Self-efficacy theory

Self-determination theory's key assumption is that the quality of motivation predicts the level of commitment and persistence when engaging in a behavior. In this context, researchers applying SDT usually differentiate between *autonomous motivation* and *external motivation*. Autonomous motivations are self-originated as they come from one's sense of volition, self-satisfaction, what the person values personally, while external motivations are more oriented toward others and the outside world as they are about complying with external demands such as avoidance of punishment, social pressure, etc. [15]. In the case of utilization of open science resources, we can hypothesize based on this theory that:

(iv) high levels of autonomous motivations (e.g., believing in the intrinsic good of open science) can drive higher levels of utilization.

Self-efficacy theory of Albert Bandura (1977) [18,41] postulates that perceived abilities to learn or perform actions at designated levels influence the motivation to engage in these behaviors, and in turn, influence the positive or negative outcome of the behaviors. Self-efficacy theory enjoys a lot of empirical supports and most show self-efficacy can be an important motivational construct that drive choices, effort, persistence, and achievement. Simply put, in the case of utilization of open science resources, high-efficacy people are more likely to persist and learn and keep up high level of motivation, and this can lead to better outcomes, which reinforce the self-efficacy sense as well as the motivation.

(v) A high level self-efficacy can support higher level of utilization of open science resources and this effect can

### The mindsponge model of value-filtering mechanisms

**Fig. 2:**
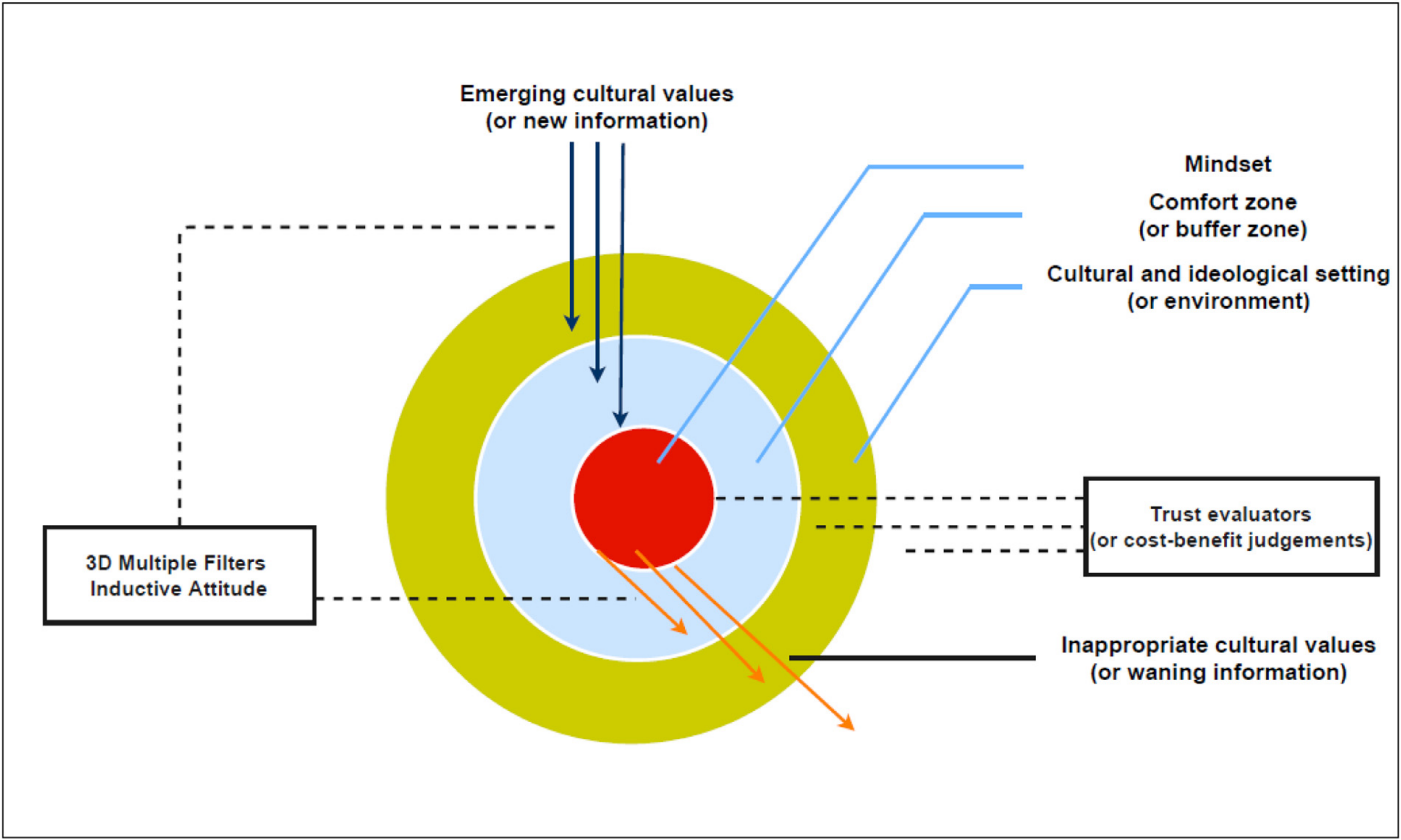
The mindsponge model for value-filtering mechanisms. Open-source license by Nguyen et al. (2021) [45].

The mindsponge model, as its name suggests, envisions the mind works like a sponge and its function is to filter new values and information [20]. This model is conceived to understand how expats and foreign businesses accept or reject new values and new innovations [20]. Unlike the presumptions in the TAM or UTAUT, in the mindsponge model, perceived costs and benefits are not primary determinants of whether a new technology or value is finally accepted [19,42]. Rather, subjective cost-and-benefits evaluation acts us trust evaluators for the mind. That is to say when the benefits outweigh the costs, a new value has gained trust and will be further processed into the core value [43,44] (see Fig. 2).

Here, an *alignmen*t with core personal, cultural, and ideological values is the *primary factor* in determining whether a new value is finally accepted and eventually becomes a part of an individual's core values set and manifest into his/her behavioral and emotional tendencies. Another prediction the mindsponge model makes is creativity, defined as the ability to creatively adapt a new value with discipline to their particular circumstances, can support the value-filtering process. Based on the mindsponge model, we can hypothesize that:

(vi) the *alignmen*t of open science values with core personal, cultural, and ideological values is the *primary determinant of its utilization*

### A synthesized questionnaire for surveying factors behind utilization of open science resources

**Fig. 3:**
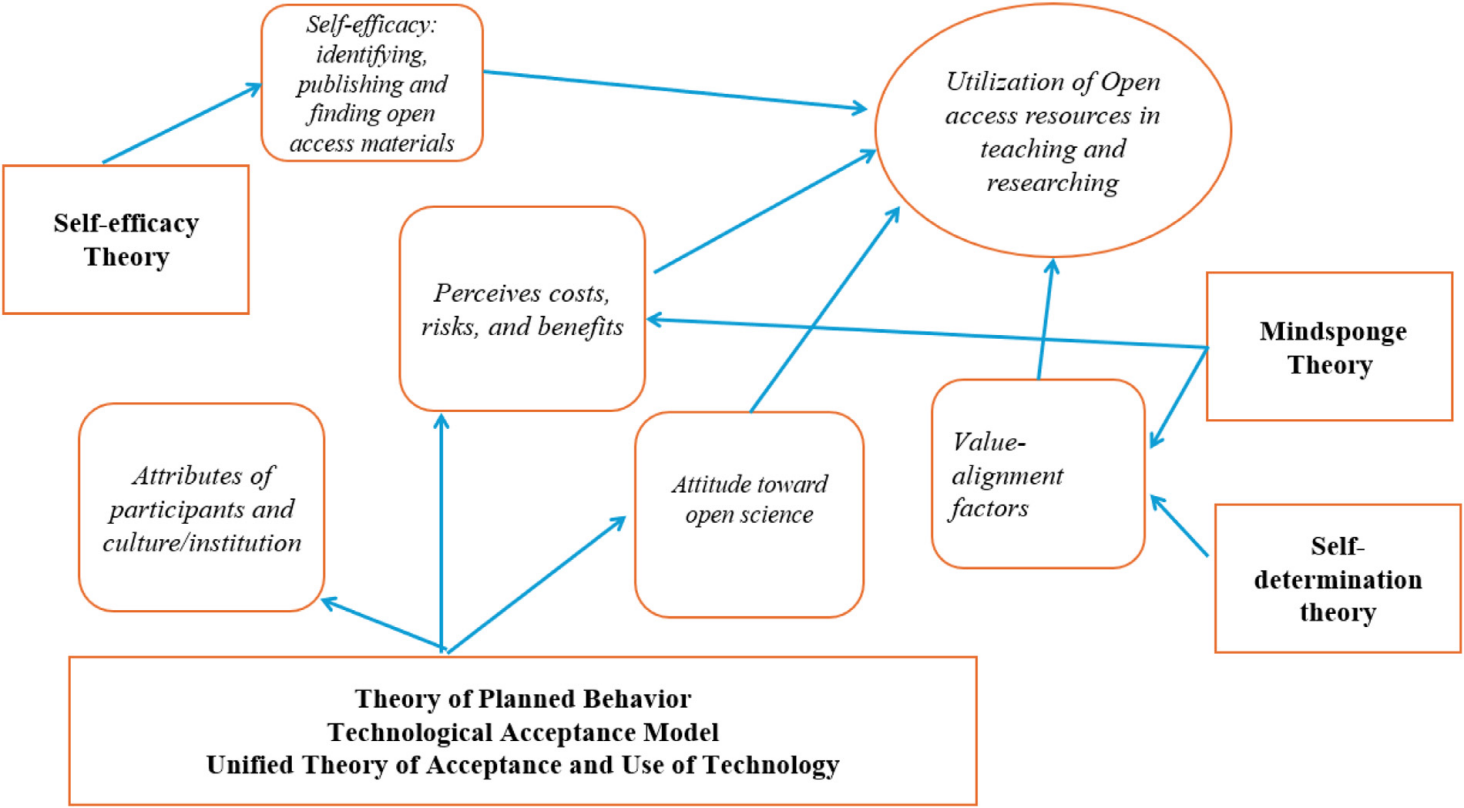
A synthesis of all methods reviewed in this study and how their key constructs contribute to a comprehensive evaluation of factors driving the utilization of open science resources.

Fig. 3 presents a synthesis of all methods reviewed so far and how key constructs from these theories can contribute to a comprehensive evaluation of factors driving utilization of open science resources. And Table 2 presents a sample questionnaire for this topic.

**Table 2:** A questionnaire for surveying factors driving the utilization of open science resources in higher education setting.

No.	Constructs	Theories	Sample survey questions for lecturers, researchers, or school staffs	Sample survey questions for university students
**Attributes of studied subjects**				
1	Age	TPB, SDT, Self-efficacy, Mindsponge TAM	Under 30 s, 30–35 years old, 36–45 years old, 46–55 years old, more than 55 years old	18–20 years old, 21–22 years old, 22–30 years old, over 30 years old
2	Sex		Male, Female	Male, Female
3	Place of residence		Rural area, suburban, cities, major cities	Rural area, suburban, cities, major cities
4	Area of expertise/ Major		Education & Pedagogy, Science & technology, humanities, etc.	Education & Pedagogy, Science & technology, humanities, etc.
5	Years of experience in teaching/ researching		Under 3 years, 3 −5 years, 5–10 years, 10–15 years, more than 15 years,	Not applicable
6	Position in organization		Entry-level staff, senior staff, management, head of school or institution	Not applicable

**Actual utilization of open resources**			Respondents choose between 1 and 7, 1 means strongly disagree and 7 means strongly agree	
7a	**For teaching**	TPB, SDT, Self-efficacy, Mindsponge TAM UTAUT	• I regularly *use or integrate* open science materials (including: OA articles, data, software/tools, open communities) into my lectures. • I regularly *guide* my students in using open science resources. • I regularly use open science materials to *create test* questions for students. • I use open science materials in meetings or discussions with other lecturers. • I regularly share my *lectures* on open science systems (e.g.: archive, OSF, openverse, ResearchGate, WikiEdu, etc.)	• I regularly use or integrate open science resources (including: public domain images, OA articles, data, software/tools, open communities) into my schoolwork. • I regularly use open science resources to support my teaching assistantship. • I utilize open science resources in meetings or discussions with other students. • I regularly share my learning materials on open science systems (e.g. archive, OSF, openverse, ResearchGate, WikiEdu)

7b	**For researching**	TPB, SDT, Self-efficacy, Mindsponge TAM UTAUT	• I regularly use or integrate open science resources (including: OA articles, data, software/tools, open communities) in reviewing literature. • I regularly use open science materials to build my methodology. • I regularly use open science materials to compare, contrast, and discuss research results. • In meetings or discussions with other researchers, I refer to open science resources. • I regularly share my preprints on open science systems (e.g.: archive, OSF, openverse, ResearchGate, WikiEdu)	• I regularly use or integrate open science resources (including: OA articles, data, software/tools, open communities) in reviewing research literature. • I regularly exploit open science resources to build my methodology. • I regularly exploit open science resources to compare, contrast, and discuss research results. • In meetings or discussions with other students, I often use open science materials. • I regularly share my essays, writings, ppts on open science systems (e.g.: archive, OSF, openverse, ResearchGate, WikiEdu)
**Capabilities and skills**				
8a	**Language skills**	Self-efficacy Theory	• I feel confident using English (reading and writing) in the field of education. • I feel confident using English (reading and writing) for academic and research purposes in general. • In addition to English, I feel confident using a third language (reading and writing) in the field of education. • In addition to English, I feel confident using a third language (reading and writing) in academic and research purposes in general.	• I feel confident using English (reading and writing) in the field of education. • I feel confident using English (reading and writing) for academic and research purposes in general. • In addition to English, I feel confident using a third language (reading and writing) in the field of education. • In addition to English, I feel confident using a third language (reading and writing) in academic and research purposes in general.

8b	**IT skills**	Self-efficacy Theory	• I feel confident in searching for academic information on the Internet. • I feel confident in sharing teaching materials, publishing my research results on the Internet. • I feel confident in publishing on the Internet even if no one guides me how to do it.	• I feel confident in searching for academic information on the Internet. • I feel confident in sharing teaching materials, publishing my research results on the Internet. • I feel confident in publishing on the Internet even if no one guides me how to do it.

8c	**Self-efficacy regarding knowledge of publishing and finding open access materials**	Self-efficacy Theory	• I feel confident about publishing open access. • I feel confident about differentiating between different forms of open access (green, gold, diamond, hybrid). • I feel confident about searching for and selecting publishers with open access journals or hybrid journals with fully open access options (e.g. searching for journals on SCImago or DOAJ, searching for books on Open Book Publishers, Open Library of Humanities). • I feel confident about identifying the type of license to use, including open licenses, when exploiting open scientific resources. • I feel confident about using and citing materials under Creative Commons (CC) licenses properly.	• I feel confident about using and citing materials in the “public domain” properly. • I feel confident about identifying the type of license to use, including open licenses, when exploiting open scientific resources. • I feel confident about using and citing materials under Creative Commons (CC) licenses properly.
8d	**Self-efficacy regarding using open data and software resources**	Self-efficacy Theory	• I feel confident about searching for data or sharing preprints in general on open access repositories (e.g. OpenDOAR, Open Access Directory, ResearchGate, arXiv, OSFPreprints) • I feel confident about searching for data or sharing preprints related to my field of study on open access repositories (e.g. OpenDOAR, Open Access Directory, ResearchGate, arXiv, OSFPreprints). • I feel confident about using data search tools (e.g. Google Data Search). • I feel confident about properly searching and exploiting datasets on open data repositories (e.g. Dataverse, Dryad, figshare, Zenodo, re3data) • I feel confident about using open research software and open source	• I feel confident about searching for data or sharing preprints in general on open access repositories (e.g. OpenDOAR, Open Access Directory, ResearchGate, arXiv, OSFPreprints) • I feel confident about searching for data or sharing preprints related to my field of study on open access repositories (e.g. OpenDOAR, Open Access Directory, ResearchGate, arXiv, OSFPreprints). • I feel confident about using data search tools (e.g. Google Data Search). • I feel confident about properly searching and exploiting datasets on open data repositories (e.g. Dataverse, Dryad, figshare, Zenodo, re3data) • I feel confident about using open research software and open source

**Attitude**				
9	Attitude regarding self Attitude regarding others and communities	TAM TPB UTAUT	• Open science resources help me become better at learning, teaching, and researching. • I wish to receive more training and education on using open science resources. • I wish more people outside of education & pedagogy field to know how to use open science resources. • I believe that using open science resources effectively is good for the scientific community and society. • I wish more people in education to know how to use open science resources.	• Open science resources help me become better at learning, teaching, and researching. • I wish to receive more training and education on using open science resources. • I wish more people in education to know how to use open science resources. • I wish more people outside of education & pedagogy field to know how to use open science resources. • I believe that using open science resources effectively is good for the scientific community and society.
**Perceived costs/risks and benefits**				
10a	Perceived ease of use Perceived financial benefits Perceived time benefits Perceived compatibility Perceived gained innovative or creative advantages	TAM TPB UTAUT Mindsponge	• I find open science resources easy to find and use. • Using open science resources helps me reduce financial costs in learning, teaching, and research. • Using open science resources helps me save time in learning, teaching, and research. • Using open science resources is highly compatible with my daily work. • Exploiting open science resources helps me have more innovative and creative ideas.	• I find open science resources easy to find and use. • Using open science resources helps me reduce financial costs in learning. • Using open science resources helps me save time in learning. • Using open science resources is highly compatible with my daily routine. • Exploiting open science resources helps me have more innovative and creative ideas.

10b	Perceived misused risks Perceived personal reputational costs Perceived organizational reputational costs Perceived risks for quality of scientific information Perceived financial costs Perceived misinformation risks	TAM TPB UTAUT Mindsponge	• I am concerned that open science resources may be misused (e.g., not properly cited, improperly analyzed, etc.) • I am concerned that using open science resources may undermine my own reputation. • I am concerned that using open science resources may undermine the reputation of my organization. • I am concerned that the proliferation of open science resources makes it more difficult to determine quality. • I am concerned that the cost of publishing open access scientific articles is too high. • With the popularity of ChatGPT, generative AIs, using open science resources may lead to misinformation, miscitation, misunderstanding of concepts, etc.	• I am concerned that open science resources may be misused (e.g., not properly cited, improperly analyzed, etc.) • I am concerned that using open science resources may undermine my own reputation. • I am concerned that using open science resources may undermine the reputation of my university. • I am concerned that the proliferation of open science resources makes it more difficult to determine quality. • I am concerned that the cost of publishing open access scientific articles is too high. • With the popularity of ChatGPT, generative AIs, using open science resources may lead to misinformation, miscitation, misunderstanding of concepts, etc.
**Environmental/** **Cultural/** **Institutional** **factors**				
11		UTAUT Self-determination theory Mindsponge	• My institution/ university has a policy to encourage students and staff to utilize open science resources. • In my field, the trend of utilizing open science resources is increasing. • In general, many people around me express interest in exploiting open science resources.	• My university actively promote and encourage students and staff to utilize open science resources. • In my major, the trend of utilizing open science resources is increasing. • In general, many people around me express interest in exploiting open science resources

**Value-alignment factors**				
12	**Consistency with one's values**	Self-determination theory Mindsponge	• Utilizing open science resources is consistent with my own views on the development trend of education and pedagogical studies. • Utilizing open science resources is consistent with my own views on the trend of international integration. • Utilizing open science resources is consistent with my own views on my own development. • I will continue to utilizing and support open science resources even if others do not support them. • Sharing and publishing articles on open science systems helps other researchers build on my research findings.	• Utilizing open science resources is consistent with my own views on the development trend of the education and pedagogical studies. • Utilizing open science resources is consistent with my own views on the trend of international integration. • Utilizing open science resources is consistent with my own views on my own development. • I will continue to utilizing and support open science resources even if others do not support them. • Sharing and publishing articles on open science systems helps other researchers build on my research findings.

Below are some key points in our synthesis. First, as shown in Table 2, the theories reviewed above have significant overlaps and it is necessary for the researchers to clearly understand the constructs and adapt the constructs from the original theories to the studied subject: utilization of open science resources.

Second, the utilization of open science resources can be measured in both how strongly the respondents rate their engagement with open science resources in both researching and teaching practices.

Third, we have tried to adopt the constructs from the original theories into the specific context of Vietnam, a developing country. Context-specific adaptation is critical for successful research studies [46]. For example, for the context of Vietnam or any countries where English is not native, language skills can present significant barriers.

Fourth, for any countries where informational infrastructure is not evenly developed, access to infrastructure as well as possessing IT skills can present significant barriers.

Fifth, we have tried to unpack multiple dimensions of subjective evaluation of costs and risks. There are many types of costs and risks related to utilization of open science resources: reputational risks, misinformation risk, or quality of information risks, etc.

### Some discussion on statistical tools

Given the complexity of technological acceptance as well as the utilization of open science resources, researchers would be wise to deliberate carefully and test many hypotheses and assumptions and approaches with empirical data. Here, it is critical to deliberate over the choice of statistical model. For example, many studies have used some forms of Structural Equation Modeling for this kind of problem [40,[47], [48], [49]], and in a systematic review, Xue et al. (2024) observed that this modeling method is preferred. However, there are alternatives. First, ordinary least square method explored in [50,51], where simple linear regression models might explain an significant amount of variance in the data. Or another method is the Hierarchical Bayesian modeling [21,52], where factors such as attributes of the participants (age, sex, income, etc.) or their core values (i.e., level of openness to experiences, belief in open science); environmental factors of culture [36,53] can form varying intercepts for Bayesian network models to deliver better estimates on the posterior distribution of predictive variables.

### Potential limitations and some recommendations

Given the amount of items in the questionnaire, it is important to acknowledge that the survey method may face several challenges, such as survey fatigue (e.g., Jeong et al. (2023) found an extra hour of surveying increasing the skipping of answers to 10–64 % [54]), social desirability bias (when respondents provide answers they think will be viewed favorably by others [55]), and acquiescence bias (the tendency to agree with survey statements rather than expressing their true views). Thus, it is advised that to address survey fatigue, researchers should limit the number of survey items and reduce the number of dimensions and constructs to a minimum to fit with the contexts and subjects [56]. To minimize social desirability and acquiescence biases, it is recommended to conduct the survey online and anonymously, reducing the potential for in-person influence. It is also recommended that other methods such as ethnography [57,58] or semi-structured interviews can be examined [59].

## Conclusion

In conclusion, the open science movement represents a pivotal shift towards democratizing scientific values such as rigor, transparency, and replicability. However, the under-utilization of open science resources highlights a pressing need for a comprehensive evaluation of the factors influencing their adoption. This article has proposed a survey-based framework that integrates various relevant factors—including capabilities, attitudes, perceived costs and benefits, cultural and institutional contexts, and alignment of values—drawn from established theories in information systems. By synthesizing insights from the Theory of Planned Behavior, Self-Determination Theory, the Unified Theory of Acceptance and Use of Technology, Self-Efficacy Theory, and the Mindsponge model, we aim to provide a nuanced understanding of how these elements interact to affect the utilization of open science resources. Ultimately, this analytical framework not only supports evidence-based policymaking in academia but also mitigates potential misunderstandings surrounding emerging practices. Embracing this comprehensive approach is essential for fostering a thriving academic environment that fully realizes the benefits of open science.

## Ethics statements

None.

## CRediT author statement

**Trinh-Le Thi Tuyet:** Conceptualization, Supervision. **Hang-Nguyen Thi Thu:** Validity, visualization. **Cuong-Le Minh:** Investigation; **Dinh-Ngo Van:** Writing -Original draft preparation; **Linh-Hoang Khanh:** Methodology, Software **Trinh-Do Thi:** Methodology, Software **Thuy Phuong Tram Nguyen:** Writing - Original draft preparation. **Ho Nguyen:** Writing - Original draft preparation. **Manh-Tung Ho:** Conceptualization, Methodology, Writing- Reviewing and Editing.

## Declaration of competing interest

The authors declare that they have no known competing financial interests or personal relationships that could have appeared to influence the work reported in this paper.
